# Nursing Practice Environment in the Armed Forces: Scoping Review

**DOI:** 10.3390/nursrep15110394

**Published:** 2025-11-07

**Authors:** Mafalda Inácio, Maria Carvalho, Ana Paulino, Patrícia Costa, Ana Rita Figueiredo, Elisabete Nunes, Paulo Cruchinho, Pedro Lucas

**Affiliations:** 1 Nursing Research Innovation and Development Centre of Lisbon (CIDNUR), Nursing School of Lisbon, 1600-190 Lisbon, Portugal; mafaldainacio@esel.pt (M.I.); mjdcarvalho@campus.esel.pt (M.C.); a.paulino@campus.esel.pt (A.P.); patriciacosta@esel.pt (P.C.); anaritafigueiredo@esel.pt (A.R.F.); pjcruchinho@esel.pt (P.C.); prlucas@esel.pt (P.L.); 2 Nursing Administration Department, Nursing School of Lisbon, Avenida Prof. Egas Moniz, 1600-190 Lisbon, Portugal; 3 Department of General Surgery and Gastroenterology, Instituto Português de Oncologia de Lisboa, 1099-023 Lisbon, Portugal

**Keywords:** work environment, nursing administration research, military nursing, leadership, scoping review

## Abstract

**Background:** The nursing practice environment is a critical determinant of healthcare quality, patient safety, and nurse well-being. Military healthcare settings present unique challenges, including rigid hierarchical structures, deployment rotations, and resource constraints, which may significantly affect the nursing practice. This scoping review mapped the available scientific evidence on the nursing practice environment in military healthcare institutions and identified its influencing factors. **Methods:** Following JBI methodology, a scoping review was conducted according to the PCC framework: nurses (Population), the nursing practice environment (Concept), and military healthcare settings (Context). Papers in English, Portuguese, or Spanish were included without date restrictions. Searches were performed in 4 databases (September 2025) and data selections were conducted independently by two reviewers. **Results:** Eleven studies (2010–2025), mainly from the United States, met the inclusion criteria. Thematic analysis revealed three main components influencing the nursing practice environment: structural (leadership, professional development, staffing), relational (collaboration, conflict management), and outcome-related (well-being, retention, patient safety). Favourable environments were associated with higher satisfaction, retention, and reduced burnout. Conversely, unfavorable environments, often influenced by rank hierarchy, deployment rotations, and organizational rigidity, were linked to turnover intention, moral distress, and compromised patient outcomes. **Conclusions:** Evidence from the included studies indicates that adaptive leadership, interprofessional collaboration, professional development and staffing adequacy are recurrent factors associated with nurses’ satisfaction, retention, and perceived quality of care. Hierarchy structures, deployments, and mobility also appear to influence the specific characteristics of military nursing practice environments.

## 1. Introduction

The nursing practice environment (NPE) is a determinant of healthcare quality, patient safety, and nurse well-being [1,2,3]. The concept of the environment, first emphasized by Florence Nightingale in the nineteenth century [4], gained research relevance in the 1980s with a growing recognition of how organizational, social, and cultural factors influence nursing practice, care quality, and job satisfaction [5,6]. Defined as the complex of physical, social, organizational, and cultural factors affecting nursing care delivery [5,7], the NPE is widely understood to influence both nursing practice and patient outcomes [5,8,9,10,11,12]. Favorable NPEs are consistently linked to lower mortality, fewer adverse events, and higher professional satisfaction [2,3,13,14,15], while unfavorable environments increase burnout, turnover, and compromised safety [2,14,16,17].

Military nursing is a professional practice enriched by military experience [18,19], requiring competencies extending beyond civilian nursing [19]. Ma et al. [20] further emphasized this by identifying four core domains that capture the unique breadth of competencies in this field: clinical nursing skills, which encompass fundamental and specialized clinical practice to ensure patient safety; military nursing skills, including combat care and the ability to work in austere and high-risk environments; professional ability, such as communication, leadership, health education, management, and research; and comprehensive quality, referring to values, motivation, resilience, and personal attributes essential for effective and ethical practice. Military nursing provides care across diverse and austere settings [17,19,21], including hospitals, primary care, aircraft, ships, combat zones, and disaster or humanitarian missions worldwide [20,22,23,24,25]. This unique scope demands clinical expertise, adaptability, and resilience. These competencies are particularly important for military nurses who frequently deploy or undergo mandatory relocations (Permanent Change in Station), requiring flexibility to adjust to new work environments, clinical settings, and organizational structures. Beyond the military domain, evidence demonstrates that the nursing practice environment itself is profoundly reshaped in conditions of armed conflict [25,26,27,28]. Civilian and volunteer nurses deployed to humanitarian crises have described working in unstable, resource-constrained, and emotionally demanding environments requiring resilience, adaptability, and interprofessional coordination [25,26]. In these contexts, the NPE becomes a dynamic and adaptive space where teamwork, creativity, and psychological endurance are essential for sustaining care [25,26,28,29].

Similarly, Muhammad and Rizek [27] described the collapse of the nursing practice environment in Gaza, where emergency nurses deliver care to displaced women amid infrastructure destruction, staff shortages, and continuous mass-casualty events [27]. Their work demonstrates how the NPE, when deprived of basic safety, sanitation, and privacy, evolves into a setting where ethical commitment, compassion, and cultural sensitivity replace conventional organizational resources. The authors underline the importance of maintaining dignity, privacy, and cultural respect despite extreme constraints—core elements of a humane practice environment even under siege [27].

Recent global conflicts further emphasize that both civilian and military nurses operate within practice environments marked by moral distress, risk exposure, and ethical dilemmas [28,29]. In the Israel–Gaza conflict, Hamama et al. [29] identified two profiles among civilian nurses, “reactive” and “resilient,” based on perceived support and psychological distress, showing how leadership and collegiality within the NPE mitigate trauma-related outcomes [29]. Likewise, Reynolds [28]) documented the experiences of Ukrainian nurses caring for patients in basements and shelters under bombardment, illustrating how the NPE becomes intertwined with moral courage, advocacy, and professional solidarity [28].

Across these diverse contexts, the NPE emerges as a unifying framework for understanding nursing practice in crisis conditions. Whether in military or civilian settings, favorable practice environments, characterized by supportive leadership, ethical guidance, and organizational trust [2,14], enhance resilience and quality of care [3,16,19,30,31,32,33], hostile or under-resourced environments intensify moral distress and professional exhaustion [25,26,27,28,29]. This multidimensional perspective reinforces the need to study the NPE in military healthcare institutions, where structural hierarchy, frequent mobility, and exposure to crisis contexts intersect to shape both nurse and patient outcomes.

Studies confirm that favorable NPEs in military contexts improve teamwork, reduce emotional exhaustion, and strengthen retention [1,33,34]. Conversely, unfavorable environments are associated with dissatisfaction, moral distress during deployments, and higher turnover intention [25,34]. Moral distress is a significant challenge for military nurses in crisis situations, as they often face ethical conflicts and difficult decisions, such as providing care with limited resources or sacrificing physical safety [19,35,36]. Within this context, the nurse manager plays a fundamental role in promoting a safe and effective practice environment [19]. Nurse managers are crucial for implementing modifiable factors that enhance the NPE, such as supportive leadership, professional development, safe staffing, and nurse participation in decision-making [37,38,39,40,41]. Transformational and relational leadership are particularly relevant in military healthcare, where rigid hierarchies may otherwise hinder collaboration [42]. However, evidence shows that during high-pressure operational contexts, such as base-wide readiness exercises, deployments, or crisis response, situational and even autocratic leadership approaches may be necessary to ensure safety, coordination, and rapid decision-making [19,43,44,45]. Effective military nurse leaders demonstrate the flexibility to adopt directive leadership when urgency requires it, while tempering this stance through transparency, clear communication, and explanation of operational needs [19,46]. This situational adaptability aligns with the principles of adaptive leadership, which have been associated with reduced burnout and enhanced resilience among military nurses [47]. Therefore, fostering hybrid leadership models that combine transformational, relational, situational, and adaptive components can strengthen teamwork, morale, and retention across military nursing environments [42,48].

While findings from civilian hospital studies on the NPE are often applicable to military nursing, important differences exist [17]. Swiger et al. [33] argue that studying the NPE in military institutions is crucial because the environment and patient outcomes can be influenced by military hierarchy, particularly the difference in rank between nurses and physicians [33]. This hierarchy may inhibit or cause insecurity for nurses in expressing concerns or opinions about patient care, especially when the physician holds a superior rank [16,17,31,49,50]. For these reasons, studying the NPE in a military context is particularly important due to its unique characteristics that impact both nurses and patients’ outcomes.

Despite the growing literature, no comprehensive scoping review has mapped the NPE in military healthcare institutions. A preliminary search was conducted to verify the existence of previous scoping or systematic reviews on this topic across various databases, including MEDLINE (via PubMed), CINAHL (via EBSCO), Cochrane Database of Systematic Reviews, JBI Evidence Synthesis, PROSPERO, Evidence for Policy and Practice Information, Open Science Framework (OSF), and Epistemonikos.

### Aims and Research Questions

The primary objective of this scoping review is to map the available scientific evidence on the nursing practice environment in military healthcare institutions. The secondary objectives are to identify and describe the factors that influence the nursing practice environment in these settings; to examine how these factors are associated with nurse and patient outcomes; to identify existing knowledge gaps and discuss implications for nursing management in military healthcare institutions. This review is guided by the following research questions: (1) What is currently known about the characteristics and influencing factors of the nursing practice environment in military healthcare settings? (2) How are these factors associated with nurse and patient outcomes?

## 2. Materials and Methods

This scoping review was conducted in accordance with the Joanna Briggs Institute methodology [51] and reported in line with the Preferred Reporting Items for Systematic Reviews and Meta-Analyses Extension for Scoping Reviews (PRISMAScR) [52]. The completed PRISMA-ScR checklist is provided as Supplementary File S1. The review’s title was registered in OSF (https://osf.io/yrauf/overview accessed on 5 October 2025).

### 2.1. Eligibility Criteria

Inclusion criteria for the articles were established according to the components of the research question, using the PCC (Population, Concept, and Context) framework [51].

- The Population (P) included nurses (either military or civilian) working in military healthcare settings.
- The Concept (C) referred to the nursing practice environment, encompassing studies that describe NPE characteristics or programs for developing healthy NPEs.
- The context was restricted to military healthcare settings, including primary care, hospital, training, pre-hospital, and combat environments. Studies conducted in a civilian context were excluded.

### 2.2. Types of Sources

This scoping review included quantitative, qualitative, and mixed-methods studies. For quantitative studies, the review considered experimental and quasi-experimental designs, such as randomized controlled trials, non-randomized controlled trials, before-and-after studies, and interrupted time-series studies. Additionally, analytical observational studies—including prospective and retrospective cohort studies, case–control studies, and analytical cross-sectional studies—were considered for inclusion. Descriptive observational study designs were also included, such as case series, individual case reports, and descriptive cross-sectional studies.

Qualitative studies that focused on qualitative data were also considered. This included, but was not limited to, designs such as phenomenology, grounded theory, ethnography, qualitative description and action research.

Additionally, systematic reviews that met the inclusion criteria were considered. The review also incorporated grey literature, including opinion articles and texts, master’s and doctoral theses and dissertations, unpublished studies and book chapters, to ensure a comprehensive overview of the available evidence.

### 2.3. Search Strategy

The search strategy aimed to locate both published and unpublished studies and was planned in three stages [51].

The first stage included a preliminary search of the MEDLINE (via PubMed) and CINAHL (via EBSCO) databases to identify the most frequently used keywords, index terms, and natural language terms. The terms initially used were “Military nurses,” “Military Personnel,” “Nursing Practice Environment,” “Work Environment,” and “Military Health Services.” Words found in the titles and abstracts, as well as the index terms used to describe the articles, were analyzed to develop a comprehensive search strategy for each database. MeSH terms and database-specific subject headings, along with Boolean operators, were used in the search strategy equations.

In the second stage, all keywords and index terms identified in each database were used. The databases, searched in September 2024 and updated in September 2025, included CINAHL, MEDLINE, Military & Government Collection, and Academic Search Complete. The search strategies are presented in Supplementary File S2. Sources of unpublished studies and gray literature were searched in the ProQuest Dissertations, Latin-American and Caribbean Health Sciences Literature (LILACS), and RCAAP databases using the same search strategy and keywords.

The reference lists of included articles were inspected for additional sources. And it was not necessary to contact the corresponding authors of the included sources of evidence.

This review was conducted by two independent reviewers and included studies published in Portuguese, English, and Spanish. No time limit was defined as the goal was to cover all available literature on the topic due to its scarcity in the military context.

### 2.4. Study Selection

Following the search, all identified citations were compiled and uploaded to the software Rayyan^®^ (https://www.rayyan.ai accessed on 5 October 2025) [53] and duplicates were removed. Although Rayyan offers optional AI-assisted features, these functions were not used in this review. All screening and eligibility decisions were performed manually and independently by the reviewers to ensure methodological rigor and transparency. After a pilot test, titles and abstracts were screened by two independent and blinded reviewers against the review’s inclusion criteria. Studies that did not meet the inclusion criteria were excluded. Potentially relevant articles were retrieved in full. The full-text evaluation of the selected citations was conducted by two independent reviewers (MI, MJ). During the various stages of the selection process, disagreements between the two reviewers were resolved without the need to consult a third reviewer (AP). The reasons for excluding sources that did not meet the inclusion criteria were recorded.

The search results and the study inclusion process were fully documented in the Preferred Reporting Items for Systematic Reviews and Meta-Analyses extension for Scoping Reviews (PRISMA-ScR) flow diagram [54].

### 2.5. Data Extraction

Data extraction was carried out using a table, following the objectives and questions of the review, and adhering to the JBI scoping review methodology [51]. The extracted information included author(s), year of publication, country, title, objectives, study design, study population, sample size, participants, context, relevant concepts from the review question, measurement instruments, and key findings. A concise summary of the included studies and their main findings is provided in Supplementary File S3. During data extraction, any disagreements between the two blinded reviewers were resolved with a third reviewer. It was not necessary to contact the authors of the primary studies for additional information.

## 3. Results

### 3.1. Source Inclusion

**Figure 1:**
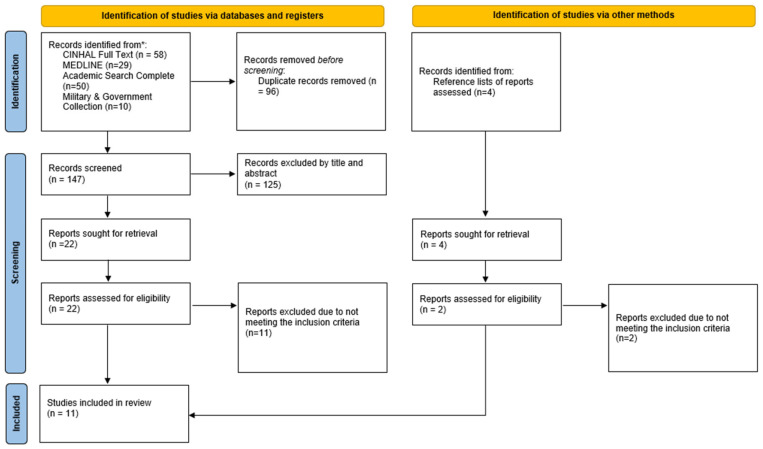
PRISMA 2020 flow diagram for new systematic reviews, which included searches of databases, registers, and other sources [54]. The asterisk indicates the databases included in the search strategy: CINAHL Full Text, MEDLINE, Academic Search Complete, and Military & Government Collection.

The searches resulted in 147 papers identified. After eliminating duplicate, 96 papers were screened. Of those screened, 74 did not meet the inclusion criteria, resulting in 22 papers being retrieved for full-text consideration. After obtaining the full-text articles, a complete reading was performed, resulting in 11 studies that met the inclusion criteria, as shown in the PRISMA Flow Diagram (Figure 1) for the study selection process.

### 3.2. Characteristics of Included Sources

The publication period ranged from 2010 to 2025, reflecting a growing interest in the nursing practice environment in military healthcare institutions over the past 15 years.

Most of the included studies were conducted in the United States of America (*n* = 8, 72.7%) [3,31,32,33,34,49,55,56]. The remaining publications originated from South Africa (*n* = 1) [49], Saudi Arabia (*n* = 1) [57], and China (*n* = 1) [30], each accounting for 9.1% of the sample.

Quantitative designs predominated, representing 77.8% of the included studies [3,30,32,33,34,49,55,56]. In addition, one qualitative study was included [58] and two systematic reviews of the literature [31,57].

### 3.3. Review Findings

The identification of the review’s findings resulted from a thematic analysis conducted in line with the study’s objective. An inductive approach, with themes emerging directly from the data, was applied in accordance with the recommendations of Peters et al. [59] and Pollock et al. [60]. Studies conducted in military institutions demonstrate that creating and maintaining a favorable nursing practice environment requires investment in structural, relational, and outcome components. Consequently, the results of the thematic analysis were mapped with the elements grouped into these three categories. Structural components correspond to the organizational and managerial conditions that shape the practice environment, such as leadership and management, opportunities for professional development and career progression, and staffing adequacy. Relational components refer to the relational and collaborative dynamics of practice, including interprofessional collaboration and conflict management. Finally, outcome components include the consequences of the practice environment, such as nurse well-being and job satisfaction, retention, quality and safety of care, and the prevention of complications versus health gains. Table 1 presents the studies included in this review, organized according to these components.

## 4. Discussion

This scoping review aimed to map the available evidence on the nursing practice environment in military healthcare contexts. The findings demonstrate that a favorable NPE, which is supported by effective leadership, adequate resources, and healthy interpersonal relationships, is consistently associated with positive outcomes for both patients and professionals [3,8,33,34,57]. Conversely, unfavorable environments, characterized by inappropriate nurse-to-patient ratios, inadequate communication, and limited resources, are associated with increased adverse events [33,55], reduced job satisfaction [34,49], and higher turnover intentions [49,55,56,57,58]. However, it is important to note that nurses’ turnover intentions may not always result in actual turnover. Constraints such as insufficient time at the current duty station, limited availability of permanent change in station opportunities, and the overriding needs of military service can delay or prevent relocation, thereby influencing workforce stability and satisfaction dynamics [19,30,42,61].

Recent evidence from both military and civilian conflict settings reinforces that leadership, collaboration, and adequate support systems are essential for maintaining effective practice environments under crisis conditions [25,26,27,29].

In addition, evidence indicates that the NPE in military contexts has unique characteristics that may influence both the validity and transferability of evaluation instruments [3,32,34,56]. Among these, the “Practice Environment Scale—Nursing Work Index (PES-NWI)” emerges as a reliable tool to evaluate the NPE, with most items demonstrating strong contributions to the overall construct.

The discussion is organized around the three components (structural, relational, and outcome) that were identified through the thematic analysis and mapping of the evidence.

### 4.1. Structural Components

#### 4.1.1. Leadership and Management

Leadership is widely recognized as a central element in creating and maintaining a favourable nursing practice environment [3,32,34,49,56]. Nurse managers play a critical role in conflict resolution, facilitating teamwork, and fostering a healthy NPE, even in contexts characterized by high diversity and strict hierarchical structures such as military institutions [34,56,58]. Nurse managers who demonstrate effective interpersonal and conflict management skills help mitigate the impact of challenging organizational conditions [32,58]. In addition to reducing conflict, effective leadership strengthens the NPE and contributes to nurse retention and satisfaction [34,58]. Conversely, ineffective or authoritarian leadership styles have been associated with professional dissatisfaction and increased workplace conflict [34]. However, during high-pressure operational contexts, such as base-wide readiness exercises, deployments, or crisis response, situational and even autocratic leadership approaches may be necessary to ensure safety, coordination, and rapid decision-making. Effective military nurse leaders demonstrate the flexibility to adopt directive leadership when urgency requires it, while tempering this stance through transparency, clear communication, and explanation of operational needs [19,47]. This ability to foster followership in demanding environments reinforces trust, role clarity, and cohesion within the nursing team. During humanitarian crises, effective leadership similarly mediates stress and supports interprofessional coordination, reinforcing the universality of leadership as a determinant of the NPE [26,27].

Evidence from military hospitals shows that favorable NPEs, fostered through supportive leadership, significantly increase nurses’ intention to remain while reducing burnout [30]. Similarly, Williams et al. [31] emphasized leadership as one of the most critical determinants of a healthy work environment, identifying transformational, relational, and authentic leadership approaches as essential for sustaining nurse engagement, job satisfaction, and patient safety.

The findings from this study align with evidence from civilian contexts, which highlights transformational and authentic leadership as protective against burnout and predictors of job satisfaction and commitment [6]. Negative leadership, conversely, has been consistently associated with dissatisfaction, conflict, and turnover across both settings [34].

#### 4.1.2. Professional Development and Career Progression

Career progression opportunities and the provision of evidence-based nursing care significantly contribute to nurses’ job satisfaction and reduce nurse turnover intention [34,56]. Therefore, professional development is a fundamental element for promoting job satisfaction while simultaneously improving the quality of care provided [56,57]. Civilian studies corroborate these findings, showing that structured professional development, mentorship, and continuing education programs strengthen retention and workforce engagement [38,40,41]. However, frequent deployments and compulsory rotations in the military may disrupt career progression, making tailored development programmes particularly important in this context [19,33].

By reinforcing autonomy and influence within the NPE, professional development contributes not only to competence but also to a more resilient, sustainable, and attractive work environment for military nurses [30]. Williams et al. [31] emphasized the importance of mentoring and shared governance as relational strategies that reinforce professional development and improve the nursing practice environment.

These findings emphasize that career progression and continuing education initiatives are not merely individual growth opportunities, but also structural and relational levers that sustain favorable practice environments in military healthcare settings [30,31].

#### 4.1.3. Staffing and Resources

A favourable nursing practice environment is intrinsically linked to professional satisfaction, and its sustainability requires the adjustment of nurse-to-patient ratios and the provision of adequate resources [56,57]. Conversely, resource scarcity and excessive workloads undermine the NPE, contributing to dissatisfaction, burnout, and increased turnover [34,57].

Staffing adequacy and resource allocation are therefore critical structural elements of the NPE, directly influencing patient safety and the efficiency of care delivery [33,56]. Poor staffing was associated with medication errors, falls, and reduced patient satisfaction [55]. Civilian evidence confirms these findings, with studies consistently linking safe staffing ratios to reduced mortality, improved safety culture, and better quality of care [8,14]. Both contexts highlight that insufficient staffing compromises quality and patient safety, though military environments face the additional challenge of resource constraints during deployment and high operational turnover [33]. Williams et al. [31] reinforced this perspective, identifying adequate staffing and resource allocation as one of the most essential recommendations for creating healthy work environments. Moreover, Jin et al. [30] (2025) demonstrated that the perception of adequate resources interacts with psychological empowerment, thereby reinforcing the overall NPE and supporting both nurse well-being and retention in military hospitals [30].

### 4.2. Relational Components

#### 4.2.1. Collaborative Practices

Interdisciplinary collaboration, particularly between nurses and physicians, is associated with greater nursing satisfaction and higher quality of care, thus contributing to a positive nursing practice environment [32,33,49,56]. Evidence from civilian settings supports this association, showing that collaborative practices are consistently linked to improved patient outcomes and higher workforce engagement [2,3,5,10]. In the military context, however, rank differences can sometimes limit open communication, potentially creating barriers not observed in civilian environments [33].

Involving nurses in clinical decision-making and fostering doctor–nurse collaboration has been shown to strengthen the NPE, reinforcing its role as a determinant of both retention and job satisfaction in military hospitals [30]. In line with this, Williams et al. [31] identified collaboration and shared governance as two of the most critical relational components of a healthy work environment. Their review emphasized that participatory decision-making not only empowers nurses but also enhances the overall climate of the practice environment, positioning collaboration as a cornerstone of structural resilience and workforce sustainability.

#### 4.2.2. Conflict Management

Organizational diversity, which is common in military contexts, is frequently described as a potential source of conflict. The literature suggests that conflicts often arise in hierarchical and inflexible environments and are exacerbated by issues such as resource scarcity and ineffective teamwork [58]. However, effective conflict management can be an opportunity to strengthen team cohesion, improve organizational performance, enrich the work environment, and promote innovation [58]. Williams et al. [31] emphasized shared governance and relational leadership as key strategies for fostering collaboration and managing conflict.

Evidence from civilian settings also confirms that conflict resolution strategies improve care outcomes, highlighting the importance of leadership and structured communication approaches in mitigating workplace tensions [37,38,39,40,41]. In military environments, however, these dynamics are intensified by the rank structure and operational stressors inherent to the context [25], which can make conflict management more complex and critical for sustaining a favorable NPE.

### 4.3. Outcome Components

#### 4.3.1. Nurse Well-Being and Job Satisfaction

Nurse professional satisfaction is a significant predictor of military nurse retention, influenced by factors such as career progression, leadership support, and compensation [34,56]. A negative nursing practice environment has a significant impact on job dissatisfaction, burnout, and the quality of care, which in turn leads to an intention to quit [3,49,57]. Civilian evidence shows similar associations, with satisfaction being the most consistent predictor of retention [38]. However, military nurses may experience additional stressors such as moral distress during combat or crisis situations, which negatively affect well-being [36]. Coping strategies, including resilience training and structured reintegration programs, have been proposed to mitigate these effects in both military and civilian contexts [22].

Favorable NPEs enhance nurses’ sense of autonomy, competence, and value, thereby improving engagement and job satisfaction while reducing burnout [30]. In this regard, Williams et al. [31] also emphasized that job satisfaction is a central outcome of healthy work environments. These findings reaffirm that the NPE is not merely a background condition but a central determinant of nurse well-being, directly shaping resilience, satisfaction, and the sustainability of the military nursing workforce [16,20,22,24,30,31,34,42]. Similarly, studies from conflict zones show that supportive environments characterized by clear communication, collegial trust, and emotional safety promote resilience and moral sustainability among nurses [27,28,29].

#### 4.3.2. Nurse Retention

Retention is a global concern in nursing, and it is consistently linked to the quality of the nursing practice environment [7,34]. Nurse retention is positively associated with professional satisfaction: the higher the satisfaction, the lower the intention to leave [34,56]. Also, civilian studies confirm this association, showing that favorable NPEs are among the strongest predictors of workforce stability across a variety of healthcare settings [56].

In military contexts, workforce stability is further challenged by frequent deployments and mandatory rotations, which can disrupt professional continuity and weaken retention [33,34]. Recent evidence indicates that while many military nurses express an intention to leave, actual attrition is frequently postponed due to contractual obligations, family stability considerations, and institutional requirements [19,30,42,61]. These dynamics illustrate how the structural and operational realities of military service can shape workforce stability and complicate retention strategies.

Jin et al. [30] demonstrated that the NPE mediates more than half of the relationship between intention to stay, empowerment, and burnout, reinforcing its central role in sustaining the military nursing workforce. Similarly, Williams et al. [31] identified retention as a key outcome of healthy work environments. In crisis or deployment scenarios, the same environmental factors mediate resilience, reducing turnover intentions [22,25].

#### 4.3.3. Patient Safety and Quality of Care

Adequate staffing and resources are essential to ensure patient safety and the efficiency of care delivery [16,56]. A favorable nursing practice environment contributes to a reduction in adverse events, such as falls and medication errors [33,55,56]. The literature also links the complexity of patient care with an increase in both medication errors and falls [55]. For example, larger hospitals with more specialized services tend to have more falls and lower patient satisfaction with care [33]. In military settings, where operational demands and resource constraints further complicate care delivery, monitoring the NPE is essential to safeguard patient safety and quality of care [3,49].

Jin et al. [30] demonstrated that improvements in the practice environment reduce burnout, indirectly enhancing the safety and quality of care. Similarly, Williams et al. [31] identified staffing adequacy, leadership, and collaboration as critical determinants of safe practice environments, emphasizing that patient safety is a direct outcome of structural and relational investments in the NPE.

These findings are consistent with a robust body of civilian literature, where supportive practice environments are associated with lower mortality, fewer adverse events, and greater patient satisfaction and nurse retention [37,38,39,40,41]. Moreover, evidence from conflict and humanitarian contexts confirms that favorable NPEs are crucial for ensuring patient safety, ethical decision-making, and continuity of care under extreme operational stress [26,31]. Studies conducted in Gaza and Ukraine demonstrate that, even amid severe adversity, strong team collaboration, moral commitment, and leadership cohesion can sustain safe and compassionate care [27,28]. These findings parallel the military evidence, reinforcing that the integrity of the nursing practice environment is a core determinant of care quality, regardless of setting.

Given these parallels, it remains essential that patient safety and quality of care in the military context be continuously monitored to minimize job dissatisfaction and improve both professional and patient outcomes [3,49].

#### 4.3.4. Prevention of Complications and Improvements in Health Outcomes

A positive nursing practice environment contributes to the reduction in adverse events, such as falls and medication errors [33,55,56]. However, the complexity of patient care can increase the occurrence of these events, indicating the need for continuous monitoring and adequate resources [49,55].

Improvements in the nursing practice environment are associated with reductions in burnout and enhanced care outcomes, indirectly preventing complications in military hospitals [30]. Likewise, healthy work environment standards—including safe staffing, leadership support, and collaboration—are directly linked to improved patient trajectories and lower complication rates. These findings confirm that strengthening the NPE is a fundamental strategy for minimizing complications and promoting health gains in military nursing practice. Civilian studies support this, linking favourable NPEs with improved recovery trajectories and reduced complications [37,38,39,40,41].

## 5. Limitations

While this review has contributed to a better understanding of the nursing practice environment in military health institutions, some limitations must be acknowledged. Although most of the included studies were conducted within the U.S. military healthcare system, several international studies also contributed to the analysis, providing valuable comparative perspectives across diverse military and healthcare contexts. However, the predominance of U.S. based research may still limit the generalizability of findings to other settings, including Portugal.

Furthermore, some of the included studies do not provide robust quantitative data regarding the direct impact of leadership and nurse-to-patient ratios on nurse outcomes. Furthermore, the cross-sectional nature of many studies prevents the analysis of the long-term impact of variables like career transitions or deployments. Future investigations, including longitudinal and mixed-method studies, are necessary to fully understand the dynamics of the NPE in military environments and its influence on professional outcomes and quality of care.

In addition, several methodological limitations should be acknowledged. Although this scoping review followed the JBI framework and PRISMA-ScR guidelines, the search strategy was limited to a predefined set of databases, which may have excluded relevant studies published. This constraint increases the potential risk of selection bias and limits the comprehensiveness of the evidence base. Moreover, the inclusion of studies with heterogeneous designs may have introduced variability in the interpretation of findings.

## 6. Implications and Future Research

The findings of this review have significant implications for nursing practice within military healthcare settings. Given the unique and demanding challenges inherent to these environments, fostering a favourable nursing practice environment is a strategic imperative. This can be achieved by prioritizing supportive and adaptive leadership [3,30,31,34,47], strengthening interdisciplinary collaboration [31,32,33,49,56], ensuring adequate staffing [8,30,33,55,56,57], providing opportunities for professional development [30,31,34,38,40,41,56,57] and career progression [19,30,33,34]. Implementing such strategies has been shown to improve nurse satisfaction [30,31,34,57], reduce burnout [6,30,31,34,47], and enhance team cohesion and retention [30,31,34,56]. Moreover, evidence from conflict and humanitarian contexts demonstrates that strong leadership, shared decision-making, and professional growth opportunities are essential to sustaining care quality and psychological resilience under crisis conditions [26,27,29]. Applying these strategies can create a positive and sustainable cycle, improving professional outcomes such as nurse satisfaction and retention, while simultaneously strengthening patient safety and the overall quality of care [3,8,30,31,33,55,56].

The findings of the present review suggest that a future systematic review would be valuable to further examine the associations between the nursing practice environment and professional outcomes in military healthcare, which may also provide valuable contributions for military nursing management. Exploring these associations in diverse cultural and organizational contexts could also enhance the global understanding of how NPE dynamics influence workforce sustainability in both military and civilian healthcare systems. Recent research underscores that civilian nurses working in conflict or humanitarian crises face challenges similar to those experienced by military nurses, including moral distress, ethical decision-making under pressure, and resource scarcity [27,28,29]. Integrating insights from these settings could inform comprehensive strategies to strengthen resilience, ethical practice, and interprofessional collaboration across the nursing discipline.

In addition, further research could explore how mandatory turnover and frequent relocations influence team cohesion, professional identity, and continuity of care within the Armed Forces. Contrasting the perceptions of nurses in managerial versus operational roles may also provide a deeper understanding of divergent experiences within the NPE. Finally, studies aimed at refining and contextualizing measurement tools for military healthcare are needed to ensure the accuracy and applicability of future assessments.

## 7. Conclusions

Military healthcare environments present unique challenges, particularly the need to reconcile rigid hierarchical structures with the promotion of collaborative and inclusive practice settings. Evidence from the included studies indicates that nurse retention in these contexts is associated with perceptions of professional development opportunities, institutional support, and overall satisfaction with the NPE.

The evidence demonstrated that a favourable nursing practice environment in military healthcare settings is commonly associated with higher nurse satisfaction and retention. This review found that supportive leadership, adequate nurse-to-patient ratios, effective teamwork, and career progression opportunities seem positively associated with professional satisfaction and negatively associated with turnover intention. Beyond these direct associations, the NPE appears to function as a mediating factor that links organizational structures and relational processes to outcomes such nurse well-being, patient safety, and quality of care.

## Figures and Tables

**Table 1:** Thematic analysis of included studies mapped to structural, relational, and outcome components of the NPE in military healthcare.

Structural components	
Leadership and management	Supportive [3,31,34,49], transformational [19,31,42] and adaptive leadership [31,42] foster engagement [3,31], teamwork [31,49], and resilience [19]. Ineffective or authoritarian styles increase conflict and dissatisfaction [3,49]. In high-pressure or deployment contexts, situational or autocratic leadership may be required [19].
Professional development and career progression	Career progression, mentorship, and ethical support mechanisms enhance motivation [31,34,57].
Staffing and resources	Adequate nurse-to-patient ratios and resource availability reduce burnout, turnover, and adverse events [16,55,57].
Relational components	
Collaborative practices	Interprofessional collaboration and collegial trust improve communication, safety, and morale [3,16,31]. Hierarchical rank structures may constrain dialogue, but structured teamwork mitigates this [19,22].
Conflict management	Multicultural and hierarchical military teams frequently experience conflict, which can be transformed into team learning through reflective and emotionally intelligent leadership [31,58]. Transparent communication and shared mission awareness reinforce followership and role clarity during deployments or readiness exercises [22].
Outcome components	
Nurse well-being and job satisfaction	Empowerment, recognition, and supportive supervision reduce burnout and emotional exhaustion, improving engagement and job satisfaction [31,49,57].
Retention and intent to stay	Favorable NPEs and ethical leadership improve retention [30,34,42,57]. However, turnover intentions may not always result in actual exits due to constraints such as deployment cycles and permanent change in station limitations [31,57].
Patient safety and quality of care	Favorable NPEs correlate with lower incidence of falls and medication errors [16,55]. Collaboration, adequate staffing, and moral commitment sustain safe and compassionate care even under crisis or war conditions [31].
Prevention of complications and health gains	Strengthening the nursing practice environment reduces adverse events and promotes measurable health gains for patients, including fewer falls, pressure injuries, and medication errors, as well as improved recovery trajectories and care continuity [16,30,31,56].

## Data Availability

No new data were created or analyzed in this study.

## Supplementary Materials

The following supporting information can be downloaded at: https://www.mdpi.com/article/10.3390/nursrep15110394/s1, Supplementary File S1. PRISMA-ScR checklist; Supplementary File S2. Detailed search strategy for each database; Supplementary File S3. Characteristics of included sources of evidence.

## Public Involvement Statement

No public involvement in any aspect of this research.

## Guidelines and Standards Statement

This manuscript was drafted against the Preferred Reporting Items for Systematic Reviews and Meta-Analyses Extension for Scoping Reviews (PRISMA-ScR) [59].
