# Supplementary material for: Nursing Practice Environment in the Armed Forces: Scoping Review
Source: Nurs Rep. 2025 Nov 7;15(11):394. doi: 10.3390/nursrep15110394 (PMC12655593; doi:10.3390/nursrep15110394)
Supplement: Supplementary file 1 [file nursrep-15-00394-s001.zip › S2_suplementary File_R.pdf]

nursing reports

MDPI

Supplementary File S2. Detailed search strategy for each database.

Interface: EBSCOhost Research Databases; CINAHL

Search	Query	Total
#1	**"Nurses" [Subject Headings] OR** "Nurs*" **OR "Military Personnel" [Subject Headings] OR** "Military Personnel" OR **"Military Nurses" [Subject Headings]** OR "Military Nurs*" OR **"Military Nursing" [Subject Headings] OR** "Armed Forces Staff" OR "Armed Forces Personnel" OR "Armed Forces Employe*" OR "Military and Naval Nursing"	1,099,078
#2	**"Work Environment" [Subject Headings] OR** "Work Environment" **OR "Health Facility Environment" [Subject Headings] OR** "Health Facility Environment" **OR** "Healthy Work Environment" OR "Nursing Workplace" OR "Workplace Environment" OR "Nursing Practice Environment" OR "Clinical Care Environment" OR "Working Conditions"	79,220
#3	**"Hospitals, Military" [Subject Headings] OR** "Hospitals, Military" **OR "Military Services" [Subject Headings] OR** "Military Services" **OR "Military Health Services" [Subject Headings] OR** "Military Health Services" OR **"Military Medicine" [Subject Headings] OR** "Military Medicine" OR "Military Facilities"	9,557
	#1 AND #2 AND #3	115
	Full text available from 2005-2025; Portuguese (0), English (58), Spanish (0)	**58**
	**Final Search Date: 24/09/2025**	

Limits applied:

- No date restrictions.
- Languages: English, Portuguese, Spanish.
- All publication types

Notes:

- Truncation (*) was used to capture plural and variant endings.
- Boolean operators (AND/OR)
- It was used controlled vocabulary (CINAHL Headings).

Interface: EBSCOhost Research Databases; Medline

Search	Query	Total
**#1**	**"Nurses"** [MeSH Terms] OR "Nurs*" [All Fields] OR **"Military Personnel"** [MeSH Terms] OR "Military Personnel" [All Fields] OR "Military Nurs*" [All Fields] OR **"Military Nursing"** [MeSH Terms] OR "Armed Forces Staff" [All Fields] OR "Armed Forces Personnel" [All Fields] OR "Armed Forces Employe*" [All Fields] OR "Military and Naval Nursing" [All Fields].	1,338,020
**#2**	"Work Environment" [All Fields] OR **"Health Facility Environment"** [MeSH Terms] OR "Health Facility Environment" [All Fields] OR "Healthy Work Environment"[All Fields] OR "Nursing Workplace" [All Fields] OR "Workplace Environment" [All Fields] OR "Nursing Practice Environment" [All Fields] OR "Clinical Care Environment" [All Fields] OR **"Working Conditions"** [MeSH Terms] OR "Working Conditions" [All Fields].	75,920
**#3**	**"Hospitals, Military"** [MeSH Terms] OR "Hospitals, Military" [All Fields] OR "Military Services"[All Fields] OR **"Military Health Services"** [MeSH Terms] OR "Military Health Services" [All Fields] OR **"Military Medicine"** [MeSH Terms] OR MH "Military Medicine" [All Fields] OR **"Military Facilities"** [MeSH Terms] OR "Military Facilities" [All Fields]	35,457
	#1 AND #2 AND #3	102
**Limiters**	Full text available from 2004<br>Portuguese (1), English (28), Spanish (0)	**29**

Limits applied:

- No date restrictions.
- Languages: English, Portuguese, Spanish.
- All publication types

Notes:

- Truncation (*) was used to capture plural and variant endings.
- Boolean operators (AND/OR)
- It was used controlled vocabulary (MEsH Terms).

Military & Government Collection

Search	Query	Total
**#1**	"Nurs*" OR "Military Personnel" OR "Military Nurs*" OR "Armed Forces Staff" OR "Armed Forces Personnel" OR "Armed Forces Employe*" OR "Military and Naval Nursing"	76,381
**#2**	"Work Environment" OR "Health Facility Environment" OR "Healthy Work Environment" OR "Nursing Workplace" OR "Workplace Environment" OR "Nursing Practice Environment" OR "Clinical Care Environment" OR "Working Conditions"	3,903
**#3**	"Hospitals, Military" OR "Military Services" OR "Military Health Services" OR MH "Military Medicine" OR "Military Facilities"	4,871
	#1 AND #2 AND #3	12
**Limiters**	Full text available from 2000<br>Portuguese (0), English (10), Spanish (0)	**10**

Academic Search Complete

Search	Query	Total
**#1**	"Nurs*" OR "Military Personnel" OR "Military Nurs*" OR "Armed Forces Staff" OR "Armed Forces Personnel" OR "Armed Forces Employe*" OR "Military and Naval Nursing"	843,001
**#2**	"Work Environment" OR "Health Facility Environment" OR "Healthy Work Environment" OR "Nursing Workplace" OR "Workplace Environment" OR "Nursing Practice Environment" OR "Clinical Care Environment" OR "Working Conditions"	94,956
**#3**	"Hospitals, Military" OR "Military Services" OR "Military Health Services" OR MH "Military Medicine" OR "Military Facilities"	13,819
	#1 AND #2 AND #3	67
**Limiters**	Full text available from 2000<br>Portuguese (0), English (10), Spanish (0)	**50**
