# Supplementary material for: Nursing Practice Environment in the Armed Forces: Scoping Review
Source: Nurs Rep. 2025 Nov 7;15(11):394. doi: 10.3390/nursrep15110394 (PMC12655593; doi:10.3390/nursrep15110394)
Supplement: Supplementary file 1 [file nursrep-15-00394-s001.zip › S3_suplementary File_R.pdf]

nursing reports

MDPI

Supplementary File S3. Characteristics of included sources of evidence – Revised

Author(s)<br>Year of publication<br>Country	Objective	Study design	Participants	Context<br>Data collection	Main results
Patrician et al. (2010)<br>USA	To examine how organizational factors, especially the nursing practice environment, influence job satisfaction, emotional exhaustion, intent to leave, and nurse-rated quality of care.	Quantitative, cross-sectional study.	Nurses (military and civilian)	Military hospital settings within the U.S. Army Medical Department	Unfavourable practice environments predicted job dissatisfaction, burnout, intent to leave, and lower quality-of-care. Leadership instability from frequent rotations reduced stability, though hierarchical respect supported teamwork. The NPE was the key determinant of nurse well-being and quality.
Koesnell et al. (2019)<br>South Africa	To explore the lived experiences of nurse managers regarding conflict management within a diverse South African military hospital and to identify implications for fostering a healthy nursing work environment.	Qualitative, exploratory, descriptive, and phenomenological study.	Military nurse managers	South African military hospital	Conflict was common in hierarchical, diverse military settings. Emotionally intelligent, inclusive leadership transformed conflict into learning opportunities, sustaining a healthy work environment.

Breckenridge-Sproat et al. (2012) USA	To examine the influence of nurse staffing levels and workload factors on unit-level medication errors and patient falls in U.S. Army hospitals, and to explore the mediating role of the nursing practice environment in these relationships.	Quantitative, longitudinal database	Nurses (active duty, reserve, civilian, and contract nurses)	Military hospital settings within the Army Medical Department	Favourable NPEs were associated with lower medication-error rates but not patient falls. Leadership, staffing, and collaboration improved patient safety regardless of ratios.
Perry et al. (2018) USA	To examine the antecedents and outcomes of nurses' self-reported job satisfaction and turnover cognitions, and to explore their relationship with patient attitudes and adverse events	Quantitative, cross-sectional study	Army Nurses	Army hospitals and clinics	Positive NPEs improved job satisfaction and patient satisfaction while reducing turnover intention. Supportive leadership, staffing adequacy, and nurse–physician collaboration were central to safer, more stable environments.
Raju et al. (2014) USA	To identify the best-fitting Item Response Theory models for evaluating the Practice Environment Scale and explore the psychometric properties of the instrument in assessing nursing practice environments.	Quantita-tive, longitudinal sudy	Military nurses	U.S. military hospitals	The Practice Environment Scale proved valid and reliable for military hospitals. Leadership engagement and communication were the strongest discriminators of positive NPEs, highlighting their importance for satisfaction.
Lang et al. (2012) USA	Examine the nursing practice environment and burnout among Army nursing personnel.	Quantitative, cross-sectional study.	Army Nurses	Combat Support Hospitals	Burnout was strongly associated with poor leadership visibility, excessive workload, and limited support.

					Supportive leadership, staffing adequacy, and teamwork promoted resilience and reduced emotional exhaustion.
Ambani et al. (2020) Saudi Arabia	To describe and compare the nursing practice environment, nurse-to-patient ratio, and job-related outcomes in a public and a military hospital in Saudi Arabia.	Quantitative, cross-sectional, correlational study employing path analysis.	Nurses (military and civil-ian)	Military hospital setting	Favourable NPEs and safe staffing predicted lower burnout and dissatisfaction and improved retention. NPE and nurse-to-patient ratio acted as mediators of job outcomes.
Swiger et al. (2018) USA	To examine the associations between dimensions of the Army nursing practice environment and patient outcomes.	Quantita-tive, longitudinal sudy	Army Nurses	Military hospital settings	Strong NPEs were linked to fewer falls and medication errors and higher patient-experience scores. Staffing/resource adequacy and nurse–physician relations were the most strongly associated with outcomes.
Zangaro et al. (2010) USA	To identify and synthesize published research assessing job satisfaction and retention among military nurses serving in the Army, Navy, and Air Force.	Systematic review	U.S. military nurses	Military healthcare settings (Army, Navy, Air Force hospitals and clinics)	Across services, supportive leadership, teamwork, and adequate resources enhanced satisfaction and intent to stay. Leadership support was the most consistent predictor of retention.
Williams et al. (2024) USA	To develop evidence-based recommendations for creating a healthy nursing	Systematic review	Military nurses	Military healthcare settings	Transformational leadership, teamwork, mentorship, and shared governance were key

	work environment to inform a professional practice model for the U.S. Military Health System				levers of positive NPEs, improving satisfaction, retention, and safety culture.
Jin et al. (2025) China	To examine the relationship between psychological empowerment and nurses' intent to stay in military hospitals, and to test the mediating effects of the nursing practice environment and burnout.	Quantitative, cross-sectional study	Military nurses	Military hospitals	Strong NPEs enhanced psychological empowerment, reduced burnout, and increased intent to stay. The NPE mediated the link between empowerment and retention.
